# Femtosecond Single Cycle Pulses Enhanced the Efficiency of High Order Harmonic Generation

**DOI:** 10.3390/mi12060610

**Published:** 2021-05-26

**Authors:** Abdelmalek Taoutioui, Hicham Agueny

**Affiliations:** 1 Institute for Nuclear Research (ATOMKI), 4026 Debrecen, Hungary; malek.taoutioui@gmail.com; 2 Physique du Rayonnement et des Interactions Laser-Matière, Faculté des Sciences, Université Moulay Ismail, Zitoune, Meknes B.P. 11201, Morocco; 3 Department of Physics and Technology, University of Bergen, Allegt. 55, N-5007 Bergen, Norway

**Keywords:** femtosecond laser pulses, coherent control, carrier-envelope phase, high-harmonic spectroscopy, high-energy plateaus

## Abstract

High-order harmonic generation is a nonlinear process that converts the gained energy during light-matter interaction into high-frequency radiation, thus resulting in the generation of coherent attosecond pulses in the XUV and soft x-ray regions. Here, we propose a control scheme for enhancing the efficiency of HHG process induced by an intense near-infrared (NIR) multi-cycle laser pulse. The scheme is based on introducing an infrared (IR) single-cycle pulse and exploiting its characteristic feature that manifests by a non-zero displacement effect to generate high-photon energy. The proposed scenario is numerically implemented on the basis of the time-dependent Schrödinger equation. In particular, we show that the combined pulses allow one to produce high-energy plateaus and that the harmonic cutoff is extended by a factor of 3 compared to the case with the NIR pulse alone. The emerged high-energy plateaus is understood as a result of a vast momentum transfer from the single-cycle field to the ionized electrons while travelling in the NIR field, thus leading to high-momentum electron recollisions. We also identify the role of the IR single-cycle field for controlling the directionality of the emitted electrons via the IR-field induced electron displacement effect. We further show that the emerged plateaus can be controlled by varying the relative carrier-envelope phase between the two pulses as well as the wavelengths. Our findings pave the way for an efficient control of light-matter interaction with the use of assisting femtosecond single-cycle fields.

## 1. Introduction

High harmonic generation (HHG) is a coherent process that plays a key role in producing ultrashort coherent light in the extreme ultraviolet (XUV) and soft x-ray range [1,2], generating attosecond pulses [3,4], and now has been growing rapidly for imaging molecular orbitals [5,6,7] and for temporal characterization of ultrafast processes [8,9].

The underlying physics of HHG is well understood on the basis of the three-step model [10], in which tunnelling, acceleration and recombination of the electrons are the fundamental steps responsible of the generation of high-order harmonics. Thus, enhancing the process relies on controlling these basic steps, and its efficiency manifests by an extension of the harmonic cutoff and/or by an increase of the harmonic yield [11]. Here, extensive theoretical works evoking different schemes have been devoted to achieve this goal. For instance, the use of chirped laser pulses [11,12,13], and the spatial inhomogeneity of the laser field [14,15] have been shown to lead to an extension of the cutoff region and or enhancing the intensity of HHG. Other schemes involving static electric fields have also been used to control HHG [16,17,18,19], although are not appropriate for time-resolved applications nor for attosecond metrology. Alternative schemes are based on the use of color mixing, in which the field-induced HHG is assisted by weaker pulses [20,21]. This includes the assisting XUV [22,23,24,25], ultraviolet (UV) [26], vacuum ultraviolet (VUV) [27] and terahertz (THz) pulses [28]. The origin of the enhancement in these color-mixing schemes was different depending on the assisting field. For instance, in the presence of a weak XUV pulse, the enhancement was linked either to the XUV-induced ac-Stark effect in the ground state [23] or to the absorption of XUV photons during the recombination step [24]. On the other hand, the enhancement in presence of a THz field was found to be caused by the modulation of the accumulated dipole phase, which results in constructive interferences of the dipole emissions (i.e., phase matching) [28].

Despite the extensive works in the field of HHG, there is still major needs for seeking new schemes capable of pushing the harmonic cutoff to higher energies with or without generating high-energy plateaus. It is thus the purpose of the present work to reveal a so far unexplored route for enhancing and controlling the HHG process, thus complementing the existing schemes and adding new insights to the general field of strong-field and attosecond physics. The proposed scheme is based on introducing a weak infrared (IR) single-cycle pulse combined with an intense laser pulse. The characteristic feature of the single-cycle pulse relies on a high-momentum transfer to electrons, leading to their displacement mainly in a single-direction. This has been discussed in the context of a THz single-cycle field interacting with Rydberg atoms [29,30] (see also [31,32,33]) and its underlying physics has been shown to be valid in the ultrafast regime [34,35], in which a coherent displacement of the electron wavepacket was demonstrated [35].

In this context, there has been recently a significant progress in developing schemes capable of producing single-cycle pulses in the infrared spectral range [36,37,38] for controlling ultrafast phenomena in gases and solids [38,39,40,41,42,43,44,45,46]. This specific interest is motivated by the properties of these pulses in isolating the electron motion in strong-light matter interaction [38]. Some application examples include the generation of high-energy electrons [47] and electron currents in the petahertz regime [48], sub-femtosecond control of the nonlinear response of bound electrons in atoms [49], precise control of the electron transport in plasmonic gaps [50], and very recently the sub-femtosecond control of freely propagating electron beam [51] was demonstrated experimentally.

It is thus timely to guide these experimental efforts and provide new insights into the role of IR single-cycle pulses in controlling coherent processes such as HHG. We aim in this work at studying to which extent the presence of a weak IR single-cycle pulse modifies the HHG process. Here, we use an intense near-infrared (NIR) multi-cycle pulse to generate high-order harmonic components and by exploiting the characteristic features of the combined pulses we show that high-energy plateaus can emerge. The emergence of this high-energy phenomenon is itself an important aspect towards establishing a new spectroscope for time-resolved electron diffraction. Specifically, we find that the characteristics of the plateau manifest by an extension of the harmonic cutoff by a factor of 3 compared to the case with only the NIR pulse. The origin of the phenomenon is linked to a displacement of the electrons caused by the single-cycle field, which in turn get further accelerated to higher momenta following a unidirectional path, thus leading to high-energy electron recollisions. We also identify the role of the field for generating even-order harmonics and controlling the directionality of the ionized electrons in the forward-backward direction. Furthermore, we show that varying the relative optical phase between the two pulses as well as the wavelength of the IR single-cycle pulse modifies dramatically the high-harmonic spectrum, and consequently, the extension of the plateaus can be controlled. Our study is based on numerical simulations of the time-dependent Schrödinger equation (TDSE). Although, the calculations are based on a one-dimensional (1D) model, the basic physics involved during the electron dynamics is verified using a three-dimensional (3D) model. Our work therefore is the first theoretical prediction of the role of a femtosecond single-cycle pulse to coherently control the HHG process. It is thus considered as a benchmark study for future theoretical and experimental works. On the other hand, our work has the advantage that the generation of femtosecond single-cycle pulses in the spectral range we consider (i.e., 5–14 μm) has been the subject of recent experimental works (e.g., [37,38]), which illustrates the feasibility of our proposed control scheme. Our findings might open up a new direction for attosecond metrology [52] for the generation of isolated attosecond pulses in the XUV and soft x-ray regions and most importantly, it might lead to a new imaging technique that combines spectroscopy and electron diffraction by exploring the high-energy electron recollisions induced by the single-cycle pulse. Our work, thus adds new insights into the coherent control of light-matter interactions [3,53,54] and ultrafast imaging [55].

This paper is organized as follows. In Section 2 we present our theoretical models based on 1D- and 3D-TDSE, including a short description of our numerical methods for solving the TDSE and for calculating the HHG spectrum. In Section 3 we present our results for HHG spectra produced by the combined pulses, and outline the effect of the presence of the single-cycle field for controlling the HHG process, and discuss the physics behind the emerged effects. The findings are supported by an analysis based on the time-evolution of the ionized electrons presented in momentum space and by the Gabor time-frequency analysis. We also discuss how the relative optical phase and the pulse wavelengths affect the HHG spectrum. Finally, in Section 4 we summarize our results on the impact of using the assisting single-cycle pulse on HHG. Atomic units (a.u.) are used throughout this paper unless otherwise specified.

## 2. Theoretical and Computational Models

**Figure 1:**
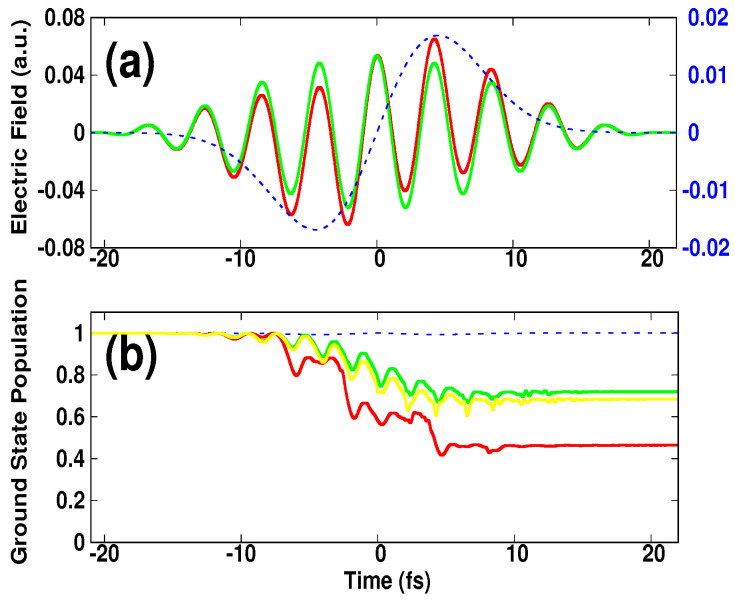
(Color online). (**a**) Laser pulses. (**b**) 1D—calculations of the temporal evolution of the population of the ground state induced by: the NIR pulse alone (green curve), the IR pulse alone (blue dashed line), and by the combined pulses (red curve). The corresponding pulses are shown in the top with the same colors. The parameters of the NIR pulse are: $\lambda_{NIR}=$ 1.27 μm, $T_{c}=$ 10 cycles, δϕ= 0 and $I_{NIR}=$ 1 × 10${}^{14}$ W/cm${}^{2}$. The parameters of the IR pulse are: $\lambda_{IR}=$ 12.7 μm and $I_{IR}=$ 1 × 10${}^{13}$ W/cm${}^{2}$. It is also shown, for reference, the population induced by only the NIR pulse, but for a peak intensity of 1.1 × 10${}^{14}$ W/cm${}^{2}$ (yellow color).

The TDSE governing the electron dynamics induced by coherent light pulses is written as

(1) $$\left[H_{0}+H_{I}\left(t\right)-i\frac{\partial }{\partial t}\right]|\psi \left(t\right)\rangle =0,$$

where $H_{0}=-\frac{\nabla^{2}}{2}+V\left(r\right)$ is the field-free Hamiltonian with the potential interaction V(r). The time-dependent interaction $H_{I}\left(t\right)$ is treated in the length gauge and is described within the dipole approximation. Without loss of generality, the electric fields are considered to be linearly polarized along the *z*-direction. Here, we consider a two-color scheme, in which the interaction $H_{I}\left(t\right)$ can be expressed as

(2) $$H_{I}\left(t\right)\;=\;-z\cdot \left(F_{\mathrm{NIR}}\left(t\right)+F_{\mathrm{IR}}\left(t\right)\right),$$

where $F_{NIR}\left(t\right)$ and $F_{IR}\left(t\right)$ describe, respectively, the NIR multi-cycle pulses and IR single-cycle pulse. In our calculations, we use the following form for the NIR pulse

(3) $$F_{NIR}\left(t\right)\;=\;E_{NIR}\cos^{2}\left(\pi t/\tau_{NIR}\right)\cos\left(\omega_{NIR}t+\delta \phi \right),$$

while the IR single-cycle pulse is expressed as

(4) $$F_{IR}\left(t\right)\;=\;E_{IR}e^{-t^{2}/\left(2\sigma_{IR}^{2}\right)}15.53\frac{t}{\tau_{IR}},$$

where $\sigma_{IR}=\tau_{IR}/4\sqrt{2\ln(2)}$ is the width of the Gaussian function in Equation (4) and *t* is defined in the range [$-\tau_{IR}/2,+\tau_{IR}/2$]. Here, $\tau_{IR}=2\pi /\omega_{IR}$, and $\tau_{NIR}=2\pi /\omega_{NIR}$ are the total duration of the IR and NIR pulses. $T_{c}$ is the total number of cycles of the NIR pulse. The parameters $\omega_{NIR}$ ($\omega_{IR}$) and $E_{NIR}$ ($E_{IR}$) are respectively, the central frequency and the amplitude of the NIR pulse (IR pulse). The amplitude $E_{i}$(i=NIR,IR) is related to the peak intensity via the relation $I_{i}=E_{i}^{2}$. The pulses in Equations (3) and (4) satisfy the condition $\int_{t_{i}}^{t_{f}}F_{i}\left(t\right)dt$ = 0, where $\tau_{i}=t_{f}-t_{i}$, and their form is depicted in Figure 1a.

We calculate the HHG spectrum H(ω) by carrying out the Fourier transform of the expectation value of the dipole acceleration along the *z*-axis

(5) $$H\left(\omega \right)\;=\;|D_{z}{\left(\omega \right)|}^{2},$$

where $D_{z}\left(\omega \right)$ is defined by

(6) $$D_{z}\left(\omega \right)\;=\;\frac{1}{\sqrt{2\pi }}\int_{-\infty }^{+\infty }<D_{z}\left(t\right)>e^{-i\omega t}dt,$$

and the time-dependent expectation value of the dipole acceleration $<D_{z}\left(t\right)>$ is written as [56]

(7) $$D_{z}\left(t\right)\;=\;\langle \psi \left(t\right)|\frac{\partial V(r)}{\partial z}|\psi \left(t\right)\rangle +F\left(t\right),$$

where F(t) is the combined NIR and IR pulses. We finally define the HHG power spectrum by

(8) $$P\left(\omega \right)\;=\;\frac{H(\omega )}{\tau_{NIR}^{2}\omega_{NIR}^{4}},$$

in which a window function of a Gaussian form $\exp\left[-{\left(t-t_{0}\right)}^{2}/\left(2\sigma^{2}\right)\right]$ centred at $t_{0}$ and having the width σ = 5.77/$\omega_{NIR}$ is used. Note that convolution with a Gaussian window allows in general a faster decaying of a desired function, which in our case is the dipole accelerator (cf. Equation (7)), at the boundaries. Its utilization prior to a Fourier transform analysis is a well-know technique that spans all physical sciences as it is useful to highlight tiny effects and has been used extensively in the context of HHG.

For further analysis we calculate the laser-induced electron current and the expectation value of the kinetic energy. These are given, respectively by

(9) $$j\left(t\right)\;=\;-ℜ[\langle \psi \left(t\right)|{\hat{p}}_{z}|\psi \left(t\right)\rangle ],$$

and

(10) $$<E>\left(t\right)\;=\;-0.5\langle \psi \left(t\right)|\frac{\partial^{2}}{\partial z^{2}}|\psi \left(t\right)\rangle .$$

The ionization wavefunction is also calculated using the same methodology as described in [57,58]

(11) $$|\psi_{ioniz}\left(t\right)\rangle \;=\;|\psi \left(t\right)\rangle -\sum_{i}|\phi_{i}\rangle \langle \phi_{i}|\psi \left(t\right)\rangle .$$

Here, the sum in Equation (11) covers the important bound states $|\phi_{i}\rangle$. We have checked that the extraction of the first 10 bound states is enough for the convergence of the ionization wavefunction.

For solving the TDSE Equation (1), we use both 1D- and 3D-models. Because of the extensive calculations involving the field range parameters, and which are performed on large spatial and temporal grids, we apt for a 1D-model. We have however verified the validity of our results by performing calculations based on a 3D-model. Therefore, the physical mechanisms discussed here hold for a realistic scenario. We stress that extensive theoretical works have been carried out using a 1D-model, and which was shown to capture the basic physics involved in an experiment.

In our numerical simulations, we consider a prototype of the hydrogen atom initially prepared in the ground state. This initial state is obtained by propagating in the imaginary time. The time evolution of the electronic wave function |ψ(t)〉, which satisfies the TDSE (cf. Equation (1)), is solved numerically using a split-operator method. In the case of a 1D-model, in which a soft potential of the form $V\left(z\right)=-1/\sqrt{z^{2}+2.0}$ is used, the method is combined with a fast Fourier transform (FFT) algorithm. For 3D calculations, the atomic potential has the form $V\left(z,\rho \right)=-1/\sqrt{z^{2}+\rho^{2}}$ and the TDSE is solved in cylindrical coordinates by combining the FFT along the *z*-axis, as in the 1D-model, and the stable Cayley transform with use of the three-point finite difference for the discretisation of the kinetic energy operator in the ρ coordinates (further details about the numerical method can be found in [57]).

The parameters of the spatial grid along the *z*-direction are kept the same for both the 1D- and 3D-model. Here, calculations are carried out in a grid of size $L_{z}$ = 8192 a.u. and $L_{\rho }$ = 1230 a.u., respectively, along the *z*- and ρ-axes, with the spacing grid dz = 0.25 a.u. and dρ = 0.15 a.u., i.e., $n_{z}$ = 32,768 and $n_{\rho }$ = 8192 grid points. The time step used in the simulation is δt = 0.02 a.u. The convergence is checked by performing additional calculations with twice the size of the box and a smaller time step. An absorbing boundary is employed to avoid artificial reflections, but without perturbing the inner part of the wave function. The boundary is chosen to span 10% of the grid size in each direction.

## 3. Results and Discussion

We considered a two-color mixing scheme implemented numerically in order to control the HHG process. The scheme consisted of an intense NIR multi-cycle pulse, which generated high-order harmonics and a weak IR single-cycle pulse introduced as an assisting field. The NIR pulse has 1.27 μm central wavelength, 42.4 fs pulse duration and 1 × 10${}^{14}$ W/cm${}^{2}$ peak intensity. The IR pulse had the same pulse duration but with a central wavelength of 12.70 μm, and the peak intensity was in the range (1 × 10${}^{10}$, 1 × 10${}^{13}$) W/cm${}^{2}$. Here, the choice of these intensities was such that the single-cycle field was introduced as a control tool and did not contribute to the ionization, but rather acted on the freely propagating electrons. This can be seen in Figure 1b (blue dashed-line), in which the temporal evolution of the ground state population was almost unchanged. At the end of the pulse, the population is 0.994, and in the case of the NIR pulse alone was 0.72, while when combining both pulses the occupation became 0.46. We also present, for reference, the population in the case the IR single-cycle pulse was replaced by a weak NIR multi-cycle pulse having the same form and the wavelength as the intense one (i.e., the total peak intensity of the NIR is 1.1 × 10${}^{14}$ W/cm${}^{2}$). The population was found to be 0.68: it showed a small depletion compared to the case when introducing the IR pulse (i.e., the induced population 0.46). Taking into consideration the characteristic feature of the single-cycle field; these preliminary results suggest that the fast depletion of the ground state might have been caused by high-energy recollision of electrons that acquired high momentum from the single-cycle field. We elaborate this discussion in the following in connection with the HHG process.

**Figure 2:**
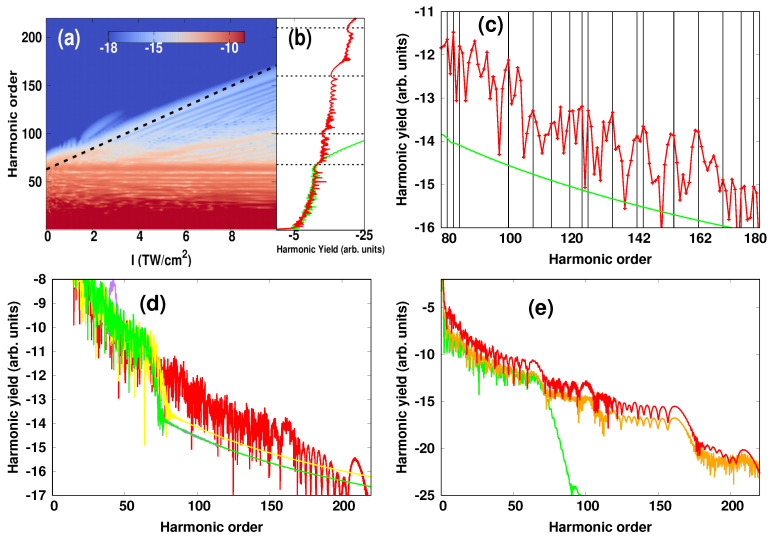
(Color online). (**a**–**d**) 1D—calculations of HHG. (**a**) Harmonic spectra as functions of the peak intensity of the IR single cycle pulse. (**b**) Harmonic spectra obtained with the assisting IR pulse (red curve) and without (green curve). (**c**) Harmonic spectrum as in (**b**) but in the harmonic order range [80:180], in which even—order harmonics are indicated by vertical lines. (**d**) Harmonic spectra obtained with different assisting fields having the same peak intensity and without using a Gaussian window: (purple curve) XUV—assisting field ($\omega_{XUV}=$ 41 eV and $\tau_{XUV}=$ 1 fs), (yellow curve) NIR—assisting field ($\omega_{NIR}=$ 0.976 eV and $\tau_{NIR}=$ 42.4 fs ) and (red curve) IR single cycle assisting field ($\omega_{IR}=$ 0.0976 eV, $\tau_{IR}=$ 42.4 fs). For reference, the spectrum obtained in the absence of the assisting field is also plotted (green curve). (**e**) 3D—calculations of the harmonic spectra as in (**b**): with the assisting IR pulse (orange curve) and without (green curve). For comparison, the spectrum obtained using the 1D—model is also shown (red curve). Yields are shown in log scale. The parameters of the NIR pulse are: $\lambda_{NIR}=$ 1.27 μm, $T_{c}=$ 10 cycles, δϕ= 0 and $I_{NIR}=$ 1 × 10${}^{14}$ W/cm${}^{2}$. The parameters of the IR pulse are: $\lambda_{IR}=$ 12.7 μm and $I_{IR}=$ 1 × 10${}^{13}$ W/cm${}^{2}$.

In Figure 2a we present the calculated HHG spectrum using the two-color scheme at different peak intensities of the IR single-cycle pulse. The calculations are based on a 1D-model. Here, the relative optical phase δϕ between the two pulses was fixed at 0. It is seen that the presence of the single-cycle pulse modified dramatically the HHG spectrum. In particular, it can be seen that the cutoff of the harmonic order $N_{c}$ increased monotonically with increasing the peak intensity of the assisting IR pulse. It extended by almost a factor of 3: It went from $N_{c}=$60 in the absence of the IR pulse to $N_{c}=$170 when the assisting pulse was introduced. The cutoff scaled linearly with the intensity $I_{IR}$, as indicated by the dashed-line in Figure 2a and was found to follow the approximative formula

(12) $$N_{c}=\left[3.17\frac{{\tilde{I}}_{IR}}{4\omega_{IR}^{2}}+E_{max}\right]/\omega_{NIR}.$$

Here, ${\tilde{I}}_{IR}=\alpha I_{IR}$ is an effective peak intensity that we consider by assuming that the single-cycle field did not reach its maximum strength when acting on the propagating free electrons. The parameter α= 0.22 is a fitting parameter and is chosen to produce the modified cutoff in the presence of the assisting field, and $E_{max}=I_{p}+3.17I_{NIR}/\left(4\omega_{NIR}^{2}\right)$ is the maximum energy that the ionized electrons can gain in the electric field of the NIR laser. The formula in Equation (12), although is simple, it illustrates the origin of the cutoff extension as a result of the excess energy (i.e., 3.17$\frac{{\tilde{I}}_{IR}}{4\omega_{IR}^{2}}$) acquired by the electrons from the IR single-cycle field, while travelling in the NIR laser field. On the other hand, we saw the emergence of multiple plateaus, which were found to be extended to higher energies when increasing the intensity of the assisting pulse. These different plateaus were visible in the 1D plot of the HHG spectrum. This is shown in Figure 2b at the intensity of the assisting IR pulse of 1 × 10${}^{13}$ W/cm${}^{2}$. The limit of each plateau was indicated by horizontal dashed lines. The first plateau, which was generated only by the intense NIR pulse was observed for $N_{c}=$ 60th, as displayed by green color. The presence of the assisting IR pulse resulted in additional plateaus: the second plateau extended up to the 100th harmonic order and was followed by a third one with a harmonic cutoff at the 160th, and even a fourth plateau which was extended up to the 210th but appeared with a weak harmonic yield.

A closer inspection of the spectrum further revealed the emergence of even-order harmonics at the high-order region as a result of including the assisting IR field. This is shown in Figure 2c, in which the even harmonics are indicated by vertical lines. In particular it is seen that odd-order harmonics were suppressed in some regions and the harmonic yield was dominated by even harmonics, and in other regions both consecutive odd and even harmonics appeared with a comparable yield. We also illustrate the particularity of introducing an IR single-cycle pulse by carrying out a comparison of HHG spectra obtained in the presence of different assisting fields. This is shown in Figure 2d, in which the assisting fields were chosen to have the same peak intensity 1 × 10${}^{13}$ W/cm${}^{2}$: XUV-assisting field ($\omega_{XUV}=$ 41 eV and $\tau_{XUV}=$ 1 fs), NIR-assisting field ($\omega_{NIR}=$ 0.976 eV and $\tau_{NIR}=$ 42.4 fs) and IR single-cycle assisting field ($\omega_{IR}=$ 0.0976 eV, $\tau_{IR}=$ 42.4 fs). This comparison shows that the presence of the IR pulse led to an increase of the harmonic yield by almost three orders of magnitude compared to other assisting fields. Note that in Figure 2c,d the spectra are displayed without using a Gaussian window, which allows a direct comparison between different assisting fields. On the other hand, the obtained results, although they were based on a 1D-model, they were found to be well reproduced in a 3D-model as shown in Figure 2e, and thus validating the 1D-based calculations.

**Figure 3:**
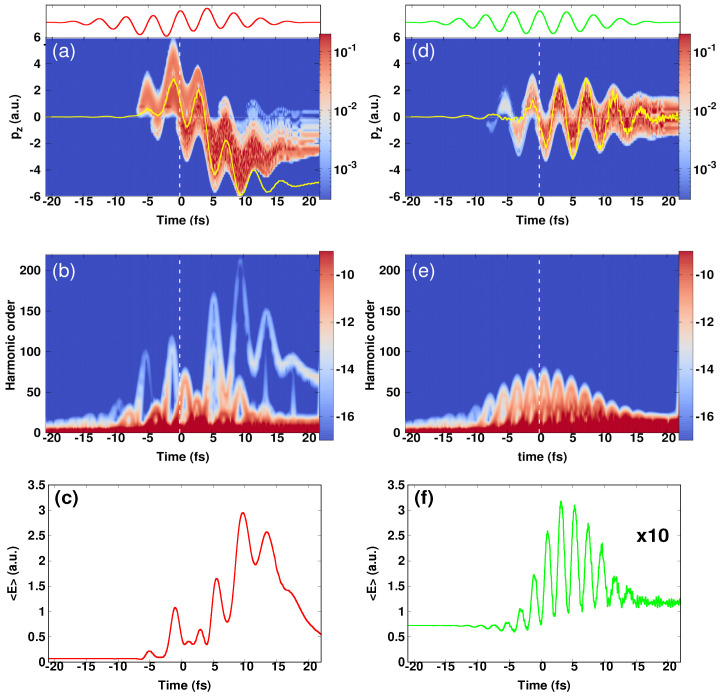
(Color online). 1D—calculations performed with only the NIR pulse (right column) and when assisted with the IR pulse (left column). Top: (**a**,**d**) Time evolution of the density of the ionized electrons. In the same figures are shown the electron currents (yellow curves). Middle: (**b**,**e**) time—frequency analysis of the harmonic spectra. The white dashed lines indicates the zero time. Bottom: (**c**,**f**) expectation value of the kinetic energy <E>. The data in Figure (**f**) are multiplied by 10 due to a weak amplitude of <E>. The parameters of the NIR pulse are: $\lambda_{NIR}=$ 1.27 μm, $T_{c}=$ 10 cycles, δϕ= 0 and $I_{NIR}=$ 1 × 10${}^{14}$ W/cm${}^{2}$. The parameters of the IR pulse are: $\lambda_{IR}=$ 12.7 μm and $I_{IR}=$ 1 × 10${}^{13}$ W/cm${}^{2}$. Insets: laser pulses taken from Figure 1a.

In order to gain more insights into the physics behind the observed effects, we evaluated the temporal evolution of the density of the ionized electrons presented in momentum space as well as the Gabor time-frequency profile [59]. These are displayed in Figure 3 for both cases: with and without the assisting single-cycle pulse. The results are shown for a peak intensity of the IR pulse of 1 × 10${}^{13}$ W/cm${}^{2}$. At a first glance, the assisting field modified dramatically the electron density in the forward-backward direction [see Figure 3a], which in turn affects the time-profile of HHG spectra [see Figure 3b]. Indeed, in the first half of the total duration of the pulse (zero-time is indicated by white dashed lines), and in the case the assisting field was introduced, one can see that most of the ionized electrons were distributed in the forward direction, while in the second half the electrons were localized in the backward direction. In both directions, the electrons were produced with high-momenta (up to 6 a.u.). In contrast to the case with the NIR pulse alone [see Figure 3d], the ionized electrons were symmetrically distributed in the forward-backward emission direction, and the maximum momentum produced was around 3 a.u.

These observations are very important in the sense that they illustrate the role of the IR single-cycle pulse in generating high-momentum electrons and controlling the directionality of the distributed electrons. The origin of the these observations can be understood in the following: during the first half-cycle of the single-cycle pulse, the released electrons by means of the intense NIR pulse received a high-momentum kick from the single-cycle field, as a result, they got accelerated while travelling in the NIR field and displaced following a unidirectional path, and predominantly ended up in the forward direction. In this direction, the electron density followed the strong oscillating NIR field, as can be seen in Figure 3a. This was also illustrated in the picture of the electron current, which here was positive, while its sign changed in the case with the NIR pulse alone, as indicated by yellow curves in Figure 3a,d. Here, the generated electrons, while acquiring high-energy from the single-cycle field, got driven by the strong NIR field to undergo multiple recollisions. The acquired energy is well described by Equation (12) and the change of the kinetic energy of the electrons could be seen in the picture of the expectation value <E>. The latter helped to measure this change with respect to the reference case, in which the assisting field was absent. The dramatic change of the kinetic energy was found to be almost nine times higher than that seen in the case with only the NIR pulse, as depicted in Figure 3c,f. Note that the <E> in Figure 3f was multiplied by 10 to make it visible.

In the time-frequency profile, the effect of introducing the assisting IR field manifests by the emergence of two emission bursts, in which the harmonic order of the strongest one was located around the 100th, and that corresponds to the maximum energy of the second plateau as depicted in Figure 2b with a horizontal dashed line. This result was supported by the calculated expectation value of the kinetic energy <E>, which showed two peaks at the same emission times provided by the Gabor profile. On the contrary, this behavior was absent in the case with the NIR pulse alone [see Figure 3e,f]. Similarly in the second half-cycle: the assisting field reversed its sign and the ionized electrons were displaced in the backward direction and acquired once again high-momenta from the assisting IR field. In this direction, these high-energy electrons were driven by the oscillating NIR field to undergo multiple recollisions. This was reflected in the time-frequency profile (see Figure 3b) by the emission of three bursts, whose harmonic orders were around the 160th, 210th and 150th, respectively in time. These bursts corresponded to the emergence of the third and the fourth plateaus, as indicated by horizontal dashed lines in Figure 2b. The signal of the fourth plateau was however weak. In contrast, only the basic harmonic components appeared in the case of the NIR pulse alone (see Figure 3e,f).

On the basis of these analyses, we concluded that the origin of the emerged high-energy plateaus was caused by the excess energy acquired by the ionized electrons from the IR single-cycle field, which then got driven by the strong oscillating NIR field to undergo high-energy electron recollisions. These findings, therefore, demonstrated the role of an IR single-cycle pulse as an attractive means for producing high-energy electrons and controlling the directionality of the distributed electrons.

**Figure 4:**
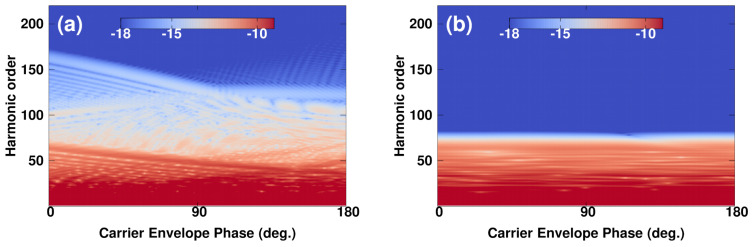
(Color online). 1D—calculations of the harmonic spectra as a function of the relative optical phase between the NIR and IR pulses. (**a**) In the presence of the assisting IR pulse and (**b**) in the absence case. The parameters of the NIR pulse are: $\lambda_{NIR}=$ 1.27 μm, $T_{c}=$ 10 cycles and $I_{NIR}=$ 1 × 10${}^{14}$ W/cm${}^{2}$. The parameters of the IR pulse are: $\lambda_{IR}=$ 12.7 μm and $I_{IR}=$ 1 × 10${}^{13}$ W/cm${}^{2}$.

Having established a comprehensive picture of the role of an IR single-cycle pulse in inducing high-energy plateaus, we now discuss how this phenomenon can be controlled. Taking advantage of the properties of the optical phase in controlling the continuum wavepacket, as it has been discussed in the context of a single-color few-cycle pulse [60] and within a two-color scheme involving two multi-cycle laser pulses [58,61], we aim here at implementing this control procedure. The basic physics involved in this control scheme is that the ionized electrons are presumed to follow the instantaneous oscillating field, as shown in Figure 3d. In general, changing the optical phase affected the time-birth of the ionized electrons, due to a time-offset expressed as $\tau =\delta \phi /\omega_{NIR}$. Therefore, electrons were generated with different final momenta depending on the optical phase, which in turn affected the maximum energy the electron gains from the laser fields. In the case of a NIR multi-cycle pulse alone, however, the final energies of the electrons were insensitive to the change of the optical phase. This is shown in the picture of the HHG spectrum [see Figure 4b], which was displayed at different optical phases covering the range [0,π]. Note that the parameters of the two-color scheme were the same as in Figure 3. When adding the IR single-cycle pulse, the spectrum exhibited a strong sensitivity to optical phase (see Figure 4a). Here, both the harmonic yield and the cutoff region were found to be modified. In particular, changing the relative optical phase by π/2 results in an enhancement of the harmonic yield. These modifications were a signature of the ultrafast coherent control of the HHG process. Thus, by varying the relative optical phase one could precisely tailor the optical cycles to yield to an unprecedented degree of control for the characteristic features of the process.

**Figure 5:**
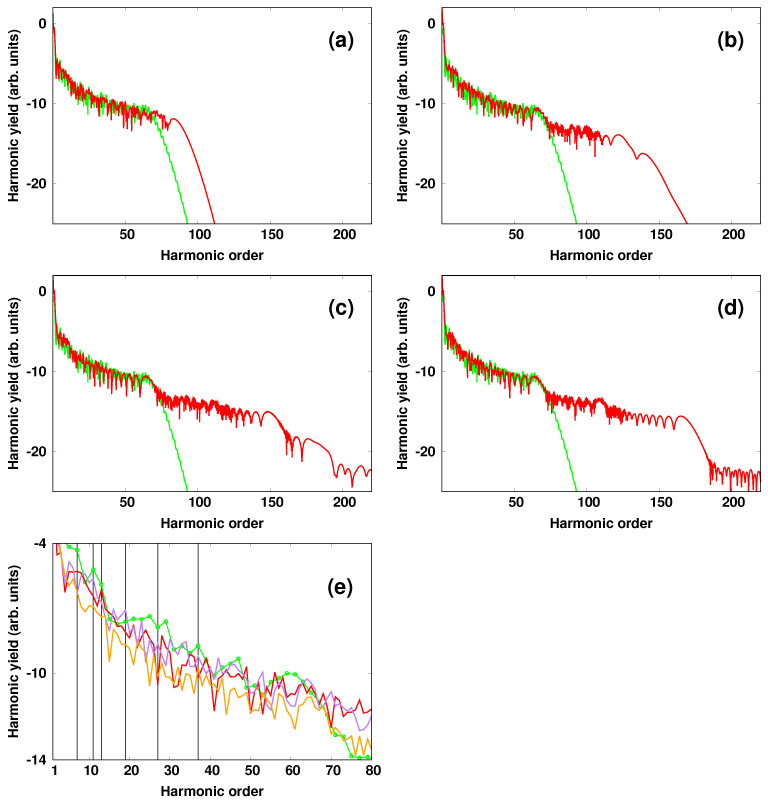
(Color online). 1D—calculations of the harmonic spectra obtained for a peak intensity of the IR single cycle pulse of 1 × 10${}^{13}$ W/cm${}^{2}$ and at different wavelengths (red curve): (**a**) $\lambda_{IR}=$$2\lambda_{NIR}$, (**b**) $\lambda_{IR}=$$4\lambda_{NIR}$, (**c**) $\lambda_{IR}=$$6\lambda_{NIR}$, and (**d**) $\lambda_{IR}=$$8\lambda_{NIR}$. For reference, the HHG spectrum obtained with the NIR pulse alone is shown with green curve in (**a**–**d**) and also with green empty circles in (**e**). A zoom of the spectrum in the harmonic order range [1:80] is shown in (**e**) for: $\lambda_{IR}=$$10\lambda_{NIR}$ (red curve), $\lambda_{IR}=$$6\lambda_{NIR}$ (purple) at 1 × 10${}^{13}$ W/cm${}^{2}$. Orange curve shows the spectrum at the IR intensity of 4 × 10${}^{13}$ W/cm${}^{2}$ ($\lambda_{IR}=$$10\lambda_{NIR}$). Vertical lines indicate the suppressed odd—order harmonics. The parameters of the NIR pulse are: $\lambda_{NIR}=$ 1.27 μm, $T_{c}=$ 10 cycles, δϕ= 0 and $I_{NIR}=$ 1 × 10${}^{14}$ W/cm${}^{2}$.

For completeness, we show in Figure 5 how tuning the wavelength of the single-cycle pulse affects the HHG spectrum. Here, we keep the parameters of the NIR pulse unchanged, and we vary both the wavelength and the peak intensity of the single-cycle pulse covering the spectral range 3–10 μm. The calculated spectra at the peak intensity of the IR pulse of 10${}^{13}$ W/cm${}^{2}$ were depicted with red color and showed an extension of the harmonic cutoff when increasing the wavelength, and that is consistent with the formula in Equation (12). Here, the harmonic order cutoff was extended from 100$\omega_{NIR}$ in the case of $\lambda_{IR}=$$2\lambda_{NIR}$ to 160$\omega_{NIR}$ for $\lambda_{IR}=$$8\lambda_{NIR}$. For reference, the HHG spectrum obtained with the NIR pulse alone was shown with green color. On the other hand, it is seen that the change of the wavelength did not affect the harmonic yield of the first plateau. A closer inspection of the spectrum, however shows some differences that emergeed on the location of the harmonic components [see Figure 5e]. In particular, the change of the wavelength from $\lambda_{IR}=$$6\lambda_{NIR}$ (purple curve) to $\lambda_{IR}=$$10\lambda_{NIR}$ (red curve) led to a suppression of some odd-order harmonics, for instance the 7th was suppressed in the case of $\lambda_{IR}=$$6\lambda_{NIR}$ and the 11th was suppressed in the case $\lambda_{IR}=$$10\lambda_{NIR}$, as indicated by vertical lines in the low-order harmonic region. For reference, odd-order harmonics in the absence of the assisting field is also shown (green curve with empty circles). On the other hand, it is seen that the change of the wavelength did not affect the harmonic yield of the first plateau. A closer inspection of the spectrum, however shows some differences that emerge on the location of the harmonic components (see Figure 5e). In particular, the change of the wavelength from $\lambda_{IR}=$$6\lambda_{NIR}$ (purple curve) to $\lambda_{IR}=$$10\lambda_{NIR}$ (red curve) led to a suppression of some odd-order harmonics, for instance the 7th was suppressed in the case of $\lambda_{IR}=$$6\lambda_{NIR}$ and the 11th was suppressed in the case $\lambda_{IR}=$$10\lambda_{NIR}$, as indicated by vertical lines in the low-order harmonic region. For reference, odd-order harmonics in the absence of the assisting field is also shown (green curve with empty circles). Insights into these observations are provided by the following expression describing the harmonic yield of a specific order *q*

(13) $$H_{q}\left(\omega_{q}\right)\;=\;|\int dz|D_{z}\left(z,\omega_{q}\right){\left|e^{-i\phi_{q}\left(z,\omega_{q}\right)}\right|}^{2}.$$

Here $|D_{z}\left(z,\omega_{q}\right)|$ is the magnitude of the dipole accelerator (cf. Equation (6)) at the point *z*, and $\phi_{q}$ is the associated phase. According to Equation (13) each harmonic $H_{q}$ resulted from a coherent sum of different dipole amplitudes $D_{z}\left(z,\omega_{q}\right)$. These amplitudes might interfere in position space either constructively or destructively depending on the phase $\phi_{q}$ (cf. Equation (13)). The presence of the single-cycle pulse might have modified this phase, and that depended on the its parameter (i.e., the peak intensity and the wavelength). The observations in Figure 5 indicate that the single-cycle pulse affected slightly slow electrons, which are the main source of the generated low order-harmonics, and thus the first plateau. These electrons felt the coulomb field and were governed by the intense NIR multi-cycle pulse. While high-energy electrons, which propagated freely, got accelerated by the single-cycle field. Those electrons were the main source for the build-up of high-energy plateaus. This picture held as the single-cycle field was not strong enough to undergo ionization. When increasing its peak intensity up to 4 × 10${}^{13}$ W/cm${}^{2}$, one can see that the harmonic yield of the first plateau got modified [see Figure 5e, orange curve]. In this case the yield diminished probably due to destructive interference effects between dipole emissions for a specific harmonics *q*.

These obtained results demonstrate that the IR single-cycle pulse affected mainly high-energy electrons, thus confirming previous experimental worked on the role of these pulses for producing electrons with higher kinetic energy [47,51] relevant for four-dimensional electron microscopy [55]. On the other hand, the results indicated that tuning the wavelength of the IR pulse allows one to control the extension of the harmonic cutoff. Therefore, the results presented in this work are valid for a wide spectral range of the IR single-cycle pulse, which demonstrates the generality of its effect on the HHG process. We have further checked that our conclusions remain valid for an intense multi-cycle laser pulse of central wavelengths in the spectral range 0.8–3.2 μm (not shown here).

At this point, our discussion presented in this work elucidates the effect of introducing a weak IR single-cycle pulse to control the HHG process, and particularly to induce high-energy electron recollisions. This is an interesting finding, since it can be exploited for time-resolved electron diffraction in an experiment, and that might lead to establishing an atomic interferometer for imaging in time and space the electron motion. With the state-of-the-art laser technology, it is possible to generate IR single-cycle pulses with peak intensities and in the spectral range discussed in our work [36,37,38,40,43,44,45,46,62]. For instance, a new scheme capable of producing relativistic single-cycle pulses of wavelength-tunable (5–14 μm) has been proposed [38,46,63]. On the other hand, it is possible to generate femtosecond NIR pulses in the spectral range 1.05–1.6 μm using an optical parametric oscillator (OPO) based on magnesium-oxide-doped periodically poled lithium niobate (MgO:PPLN) [64].

## 4. Conclusions

To conclude, on the basis of numerical simulations of the 1D-TDSE, which we verify using a 3D-model, we show that the HHG process induced by an intense near-infrared multi-cycle pulse can be controlled efficiently using an infrared single-cycle pulse. This control scheme manifests in the HHG spectrum by the generation of even-order harmonics and by an extension of the harmonic cutoff by a factor of 3 accompanied with the appearance of high-energy plateaus. The origin of this emerged plateaus is found to be related to a vast momentum kick that ionized electrons receive from the single-cycle field. These electrons in turn get accelerated to high-momenta and displaced following a unidirectional path, thus leading to high-energy electron recollisions. We also identify the role of the single-cycle field in controlling the directionality of the released electrons, which are found to be displaced mainly in the backward direction at the end of the pulses. Furthermore, it is found that these emerged effects exhibit a strong sensitivity to the change of the relative optical phase between the two pulses as well as the wavelength of both pulses. This sensitivity is shown to be a signature of ultrafast coherent control of the HHG process. Our study is not limited to atomic hydrogen as investigated here but rather can be extended to nanostructures. This extension should take into account the electronic band structure of a solid by employing effective potential models that have the capability of describing properly interband and intraband mechanisms [65,66]. These mechanisms constitute the origin of solid-state HHG process, although they contribute differently, thus rendering the process more complicated. On the other hand, the use of atomic potential based models to build up the potential interaction of a nanostructure has been shown to provide new insights into the solid-state HHG as has been shown in recent works [67,68]. Our findings establish a new control scheme which might open up a new route for precise control of light-induced nonlinear processes with the use of a femtosecond single-cycle assisting field.

## Data Availability

The data presented in this study are available on request from the corresponding author.
